# Do chimpanzees (*Pan troglodytes*) console a bereaved mother?

**DOI:** 10.1007/s10329-019-00752-x

**Published:** 2019-09-04

**Authors:** Zoë Goldsborough, Edwin J. C. van Leeuwen, Kayla W. T. Kolff, Frans B. M. de Waal, Christine E. Webb

**Affiliations:** 1 Department of Biology, Utrecht University, Utrecht, The Netherlands; 2 Behavioral Ecology and Ecophysiology Group, Department of Biology, University of Antwerp, Antwerp, Belgium; 3 Centre for Research and Conservation, Royal Zoological Society of Antwerp, Antwerp, Belgium; 4 Max Planck Institute for Psycholinguistics, Nijmegen, The Netherlands; 5 Department of Psychology, Emory University, Atlanta, USA; 6 Department of Human Evolutionary Biology, Harvard University, Cambridge, USA

**Keywords:** Thanatology, Consolation, Empathy, Bereavement, Chimpanzees

## Abstract

Comparative thanatology encompasses the study of death-related responses in non-human animals and aspires to elucidate the evolutionary origins of human behavior in the context of death. Many reports have revealed that humans are not the only species affected by the death of group members. Non-human primates in particular show behaviors such as congregating around the deceased, carrying the corpse for prolonged periods of time (predominantly mothers carrying dead infants), and inspecting the corpse for signs of life. Here, we extend the focus on death-related responses in non-human animals by exploring whether chimpanzees are inclined to console the bereaved: the individual(s) most closely associated with the deceased. We report a case in which a chimpanzee (*Pan troglodytes*) mother experienced the loss of her fully developed infant (presumed stillborn). Using observational data to compare the group members’ behavior before and after the death, we found that a substantial number of group members selectively increased their affiliative expressions toward the bereaved mother. Moreover, on the day of the death, we observed heightened expressions of species-typical reassurance behaviors toward the bereaved mother. After ruling out several alternative explanations, we propose that many of the chimpanzees consoled the bereaved mother by means of affiliative and selective empathetic expressions.

**Electronic supplementary material:**

The online version of this article (10.1007/s10329-019-00752-x) contains supplementary material, which is available to authorized users.

## Introduction

Thanatology is the study of death and dying, including associated behavioral and psychological coping mechanisms. While historically focused on humans, recently, thanatology has also been recognized as a fruitful comparative framework in relation to the sociality of other animals (Anderson et al. 2018). Given their close evolutionary relatedness to humans, chimpanzees are of particular interest for the comparative thanatological approach—by comparing humans’ and chimpanzees’ death responses, we may be able to infer the ancestral forms of death-related expressions and the extent to which these expressions persist in extant species.

The evidence from qualitative reports suggests that chimpanzees are affected by the death of a group member (e.g., Teleki 1973; Boesch and Boesch-Achermann 2000; Stewart et al. 2012), investigate the dead body of a close social partner to “make sense” of the irreversible change (Anderson et al. 2010; Cronin et al. 2011), and may tend to the dying by means of “compassionate” expressions (Boesch and Boesch-Achermann 2000: chimpanzees seek close proximity and exert soft calls to badly injured and dying group members). A further example is provided by de Waal (2014, p. 25) of chimpanzees actively caring for a dying member of their group. Moreover, chimpanzees respond with more behavioral elaboration to the death of a socially active member (adolescent) compared to the death of infants (van Leeuwen et al. 2016), although mothers may form an enduring attachment to their offspring even in the absence of a socialization period (Kooriyama 2009). Furthermore, not only chimpanzee mothers but also unrelated group members respond to the deaths of infants (Biro et al. 2010; Cronin et al. 2011), suggesting that death can have an impact on the community at large.

An important, unaddressed question concerns the possibility of selective responses toward group members that are most intrusively confronted with the death—i.e., individuals commonly referred to as “bereaved” in human societies. In both humans and chimpanzees, individuals may initially respond to the deceased individual itself, possibly reflecting the emotional bond that was shared in life between the deceased and bereaved. In human societies, there may also be extended forms of compassion directed to those deprived of a close relation through the death, such as kin and friends of the deceased. Bereaved individuals often receive increased social support and contact from their community upon losing a close social partner (Aoun et al. 2018). The question of whether chimpanzees may change their behavior toward a bereaved group member is valuable to address, for it could elucidate shared evolutionary origins of such a response.

Here, we present an analysis of behavioral responses in a captive group of chimpanzees after a recently integrated adult female gave birth to a fully developed and healthy-looking stillborn (hereafter referred to as “the event”). The event occurred during an intensive study period in which all group members were systematically observed by means of standardized techniques for over 6 months. The resultant data set allowed us to test a novel hypothesis with respect to death-related social dynamics in non-human animals: chimpanzees’ death responses may extend from the deceased to include the bereaved, possibly through empathetic expressions. To test our hypothesis, we focused on pre- vs. post-event comparisons of all group members’ affiliative interactions with the bereaved mother, as well as amongst themselves. To elucidate the nature and specificity of chimpanzees’ responses, we additionally focused on species-typical reassurance behaviors such as kisses and embraces (de Waal and van Roosmalen 1979; Fraser and Aureli 2008). Furthermore, we investigated several situational determinants to rule out possible alternative explanations. The loss of an infant arguably provides the clearest opportunistic study case wherein a bereaved group member—the mother—is readily identifiable. Despite being a one-time event, by applying a systematic approach to our observations, we may discover new aspects of chimpanzee sociality and thereby further contribute to broadening the scope of comparative thanatology.

## Methods

### Study site and subjects

Behavioral observations were conducted on 15 adult chimpanzees—11 females and four males—housed socially at Royal Burgers’ Zoo in Arnhem, the Netherlands (Table 1 for subject demographics). Two females in the sample, Erika and Moni, were added to the colony on 12-02-2016, along with a third adult female who died before the beginning of the current study period (on 14-03-2017). After an introduction process during which the colony was kept in subgroups of varying sizes, the full group was reunited on 18-10-2017 (i.e., 1 month before the current study began).

**Table 1:** Subject demographics as of the start of data collection (20-11-2017)

Individual	Sex	Age	Rank
Giambo	M	29	High^a^
Jing	M	37	High^b^
Ghineau	M	13	High
Fons	M	43	High
Raimee	F	19	High
Roosje	F	39	High
Morami	F	31	Medium
Tushi	F	26	Medium
Gaby	F	34	Medium
Moniek	F	41	Medium
Geisha	F	25	Medium
Erika^c^	F	26	Medium
Jimmie	F	58	Low
Moni^c^	F	29	Low
Tesua	F	32	Low

^a^Alpha male

^b^Beta male

^c^Recently introduced

The chimpanzees’ housing consisted of an indoor and outdoor enclosure, measuring ± 386 m^2^ and ± 7000 m^2^, respectively. During scheduled feeding and routine transfers from the indoor to the outdoor area, the chimpanzees were temporarily held in a series of smaller enclosures, not visible to the public and hereafter referred to as the ‘backstage’ cages. The design of the outdoor enclosure was semi-natural, and both enclosures were enriched with various climbing structures. The chimpanzees were fed approximately three times daily and had access to water ad libitum.

### Data collection

Two observers collected data from 20-11-2017 until 01-06-2018, 4 days a week between 9:00 and 17:00. Daily data collection protocols comprised: a 90-min group observation, 10-min focal observations (one per individual), and ad libitum sampling of agonistic interactions. During group and focal observations, all occurrences of affiliative interactions were recorded (Martin and Bateson 2007), including the initiator, recipient, and type of affiliative behavior (ethogram in Table 2).

**Table 2:** Ethogram of affiliative contacts used in the current study

Behavior	Definition
Grooming	Using hands, feet, or mouth to rake through the hair of another individual and remove particles from around the hair or the skin; may include plucking ≥ 5-s duration, new event after 30 s without grooming
Mutual grooming	Grooming another individual simultaneously as that individual grooms them; may include plucking
Body kissing	Kissing or soft gnawing on the body, or buying face in the other’s hair; excluding play
Embracing	Placing arms around the body of the other individual
Mouth–mouth kissing	Kissing in the mouth–nose region
Finger/hand in mouth	Extending a bent hand or finger to the other individual, who places the open mouth on the hand or finger

The order of focal subjects was randomized, and both focal and group samples were counterbalanced across days and observers. Two observers were concurrently trained until a Cohen’s kappa value higher than 0.70 was obtained for all observational protocols (0.79 and 0.76 for group and focal observations, respectively) before the data collection period began. Observers always collected data as a pair, with one individual observing, using binoculars if necessary, and the other recording.

### The event

On 30-01-2018, 2 months after the study period began, the observers arrived at the zoo shortly before visitor opening hours (at 9:00) and observed Moni holding a dead infant. There was no strong prior indication of Moni’s pregnancy, but physical and behavioral cues on the day of the event (i.e., blood on Moni’s vulva, proximity to and handling of the corpse) indicated that it was most likely hers, which was later confirmed by DNA analysis. The infant was a fully developed male, who appeared to be in good physical condition aside from bite marks on his chest and a large gash on the top of his head. The keepers were alerted, and the chimpanzees were moved to their backstage enclosures, where they remained for the rest of the day. Initially, Moni and the infant’s corpse were kept separate from the rest of the colony as the keepers examined them. Note that the chimpanzees were accustomed to the backstage enclosures and to being separated from the group as part of routine zoo husbandry/management protocol (e.g., to receive additional feeding).

Following the initial examination, other group members were given access to both Moni and the corpse (at approximately 10:00), upon which the observers began collecting data. Data collection on this day deviated from the normal protocol in that all behaviors involving Moni and Fons (the presumed father of the infant, based on the monitored breeding program) were continuously recorded along with proximity data. Additionally, all interactions between other group members and the corpse were recorded. Photographs and videos were taken of notable interactions. Data were collected until 15:45 that day, at which point an unsuccessful attempt was made by the keepers to separate Moni from her infant’s corpse.

**Fig. 1:**
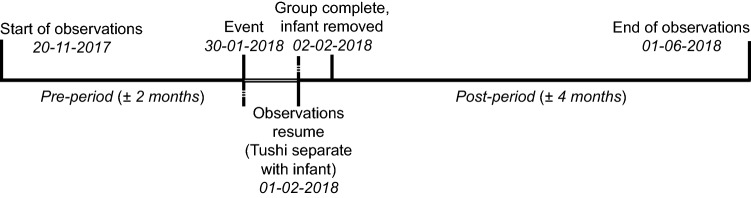
Timeline of events described in the current study, on 30-01-2018 observations occurred outside of the regular schedule and no observations took place on 31-01-2018. Observations resumed on 01-02-2018, excluding Tushi who was held in the backstage enclosures with the infant’s corpse. On 02-02-2018, the infant’s corpse was removed and Tushi reunited with the rest of the group

The next day (31-01-2018), no observations were scheduled while the chimpanzees remained in the backstage enclosure. On 01-02-2018, following several attempts on the part of the keepers to separate Moni from the corpse, it was appropriated by another female, Tushi. The rest of the group was given access to the indoor enclosure, where normal observations continued while Tushi and the corpse remained in the backstage enclosure. Videos and observations were taken of Tushi while she had the infant. At the end of this day, the group was reunited with Tushi and the corpse. The following day (02-02-2018), the corpse was dropped by Tushi and removed from the group by the keepers. It was examined by the zoo’s veterinarian, who concluded that the infant was stillborn, based on no signs of air ever having been present in the lungs. Thus, despite the extensive wounds on the body (indicating aggression towards the corpse), infanticide was ruled out as the cause of death. Subsequent DNA testing showed that Ghineau, rather than Fons, was the dead infant’s father. A more detailed description of the event can be found in Online Resource 1, and an overview of the timeline is illustrated in Fig. 1.

### Data analyses

Our analyses aimed to assess whether other chimpanzees increased their affiliation toward the bereaved mother, Moni. First, we analyzed data across all individuals to determine whether there were any subject-level differences in receipt of affiliation over time. In the case of significant between-individual variation, we would subsequently inspect these differences to compare the bereaved mother’s received affiliation trajectory to that of the other chimpanzees. This approach allowed us to determine whether changes in affiliation around the event were specific to the bereaved mother or more general. This multiple regression framework allowed us to account for several alternative factors that could potentially underlie any observed changes in affiliative patterns.

Because pregnant chimpanzees receive less affiliation (Nishida 1979) while swollen females tend to receive more (Wallis and Lemmon 1986), we added female estrus state to our overall analytical approach. Further, because the colony was outdoors more frequently than indoors in the post-period compared to the pre-period (due to warmer seasonal weather), which could impact affiliation rates, we included location of observation. Additionally, due to the introduction program that preceded the study period, it is possible that any increase in affiliation could reflect the natural integration process (Schel et al. 2013); therefore we specifically compared behavior received by Moni to that received by Erika, the other newly introduced female.

### Model specifications

First, we investigated affiliation patterns within the group (i.e., group analysis) across the study period by means of a generalized linear mixed model (GLMM; Baayen 2008) with a Poisson error distribution and a log link function in the R statistical environment v3.5.2 (R Core Team 2018). Data were structured dyadically (representing all possible actor–recipient dyads) per day, extracting corresponding given–received affiliation information from both partners’ focals and the daily group observation. The predictors of individuals’ “received affiliation” (i.e., counts per day) comprised the estrus state (i.e., in estrus vs. not in estrus vs. pregnant), age, sex, and rank of both the actor and recipient, and the location of observations (indoors or outdoors) as fixed effects. Daily observations were typically restricted to indoors or outdoors, but in some cases (< 10%) they were conducted in both enclosures; in these instances, the location in which most observations occurred was used. Further, we fitted the actor, recipient, dyad, and date as random intercepts, and “dyadic observation time” as an offset term (to account for different observation durations). To assess whether chimpanzees exhibited a differential trajectory of received affiliation over time, we incorporated random slopes of date nested in recipient. Given our interest in a (temporary) oscillation of received affiliation toward the bereaved mother, here, we allowed for a non-linear (i.e., sigmoidal) effect of time on received affiliation per individual by implementing the squared term of date nested in recipient (i.e., “1+date+I(date^2)||recipient”). Finding that this term significantly explained variance in individuals’ received affiliation would allow for a more in-depth exploration of the shapes of individuals’ trajectories, and specifically an investigation of the bereaved mother’s hypothesized increase in received affiliation immediately following the event. The overdispersion parameter of the model (i.e., 1.14) was acceptable such that the model output could be validly interpreted (threshold ~ 1.20; Payne et al. 2018). Model stability was good, as indicated by the range of estimates obtained when excluding individuals one at a time (Online Resource 2: Fig. 5).

Second, we explored the specific determinants of received affiliation for the bereaved mother in relation to other group members by running separate Poisson models with a log link function for every individual as the recipient of the affiliation (i.e., individual analyses). In order to avoid overfitting, we included only the fixed effects that pertained directly to the key potential alternatives under study (“estrus state” and “location of observation”). The same random effects structure as in the group analysis was fitted, except for the random intercept term for the recipient (i.e., here constrained to one individual). For each individual receiver, the model predictions were plotted to the actual received affiliation per month (note that since only 1 day was observed in June, this was included in May for plotting purposes). Additionally, we considered differences in received affiliation from the month pre-event (January) to the month post-event (February), to determine whether the bereaved mother’s observed increase was relatively larger than that of other group members. In order to identify how many and which group members drove the bereaved mother’s pattern of received affiliation, we also examined the results of her model per actor.

Inferences of statistical significance were based on *P* values (< 0.05) as extracted from model comparisons (with and without predictor of interest; Barr et al. 2013) using the likelihood-ratio test (Dobson 2002). Given that our hypothesis in the group analysis concerned the test of one particular term (namely the random slopes term of date squared nested in the recipient of affiliation, i.e., “1+date+I(date^2)||recipient”), we refrained from testing a full-null model comparison, where the null model would represent the absence of all fixed terms except the intercept. For the individual analysis focused on the bereaved mother, we did precede inspection of the individual effects by a full-null model comparison (Forstmeier and Schielzeth 2011), where the null model only included the intercept (set to 1).

### Qualitative data

Lastly, more descriptively, we considered the quality of the affiliative behaviors received and given by the bereaved mother on and around the day of the event, focusing on several affiliative behaviors that are typical of reassurance contexts in chimpanzees, namely body kiss, embrace, mouth–mouth kiss, and finger/hand in mouth (Fig. 3, Fraser and Aureli 2008; Goodall 1989).

## Results

In total, we collected 120 h of group observations and a mean of 13.6 h (SD 0.32) per individual of focal observations. On the day of the event, we collected 4.8 h of data focused on the bereaved mother (Moni), the presumed sire (Fons), and the infant’s corpse.

### Group analysis

**Fig. 2:**
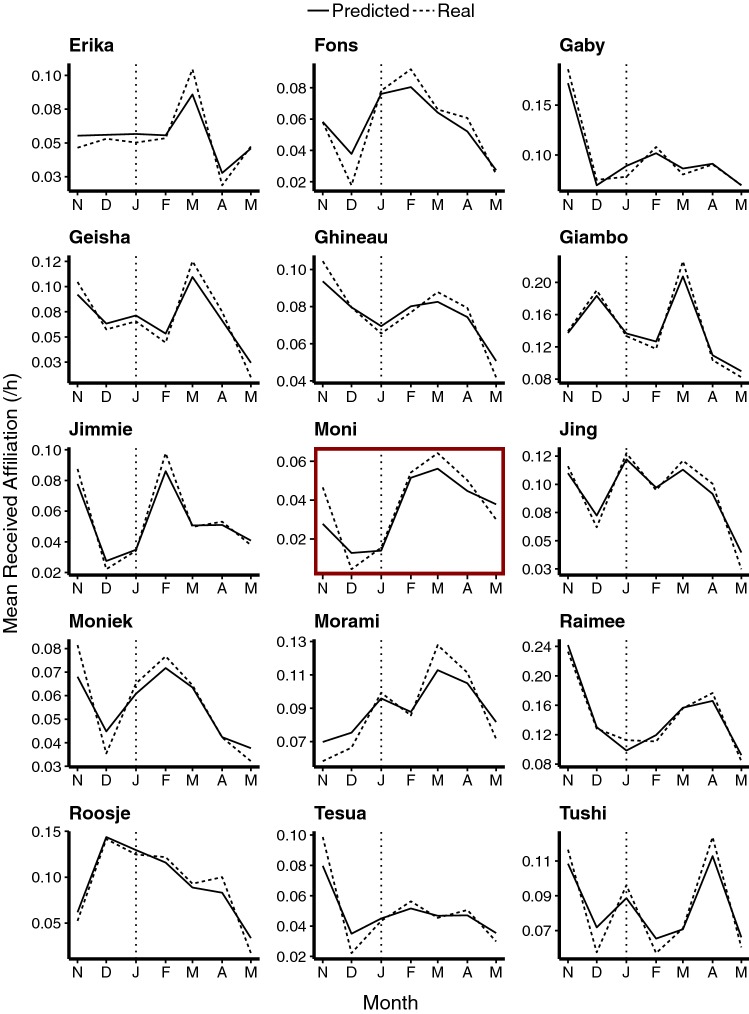
Mean hourly rates of received affiliation predicted by the GLMM (accounting for estrus state and location of observations) plotted per month and contrasted to the actual received affiliation. The vertical line indicates the stillbirth of the infant

The non-linear effect of time on received affiliation was significantly different across individuals (*χ*^2^ = 10.091, *df* = 1, *P* = 0.0015; see Fig. 2). Furthermore, at group level, the estrus state of the recipient of affiliation positively predicted the frequency of received affiliation, with pregnant females (i.e., Moni) receiving less and swollen females receiving more affiliation than non-swollen females (*χ*^2^ = 127.770, *df* = 2, *P* < 0.0001; estimate ± SD for pregnant: − 0.812 ± 0.321; swollen: 0.757 ± 0.887). Subjects received more affiliation from younger individuals (*χ*^2^ = 6.642, *df* = 1, *P* = 0.010; estimate ± SD = − 0.026 ± 0.009). No other fixed effects (i.e., actor estrus state, sex, rank; recipient age, sex, rank; observation location) significantly predicted the frequency of received affiliation (all NS; for more details on the model output, see Online Resource 2: Table 3).

### Individual analyses

Moni’s full-null model comparison was significant (*χ*^2^ = 9.45, *df* = 3, *P* = 0.024), allowing for further inspection of the individual parameters fitted. The affiliation received by Moni was significantly predicted by her estrus state (*χ*^2^ = 8.83, *df* = 2, *P* = 0.012). When pregnant, she received less affiliation than when not swollen, and there were no significant differences in her received affiliation when she was swollen compared to not swollen (estimate ± SD for pregnant: − 1.273 ± 0.493; swollen: 0.386 ± 0.429). Notably, Moni only became swollen for the first time again on 26-03-2018, nearly 2 months post-event. Observation location did not significantly predict Moni’s received affiliation (*χ*^2^ = 0.512, *df* = 1, *P* = 0.475).

Moni’s and Erika’s curves exhibited unique trajectories (Fig. 2): while Erika’s received affiliation remained stable in the months pre- and post-event, it increased sharply 2 months after the event. Moni also showed a peak at this point, but her most marked increase occurred from the month directly before the event to the month directly after the event. The distinct trajectories exhibited by these two newly introduced females helps to rule out a possible alternative explanation based on group integration. This position is further supported by rates of contact aggression and support directed towards Moni and Erika over the study period (Online Resource 2: Table 4). There was no clear decrease in aggression towards either female over time, and the month after the event was when Moni’s peak month for receiving aggression. The number of aggressive conflicts in which these females received support also remained relatively stable over time.

**Fig. 3:**
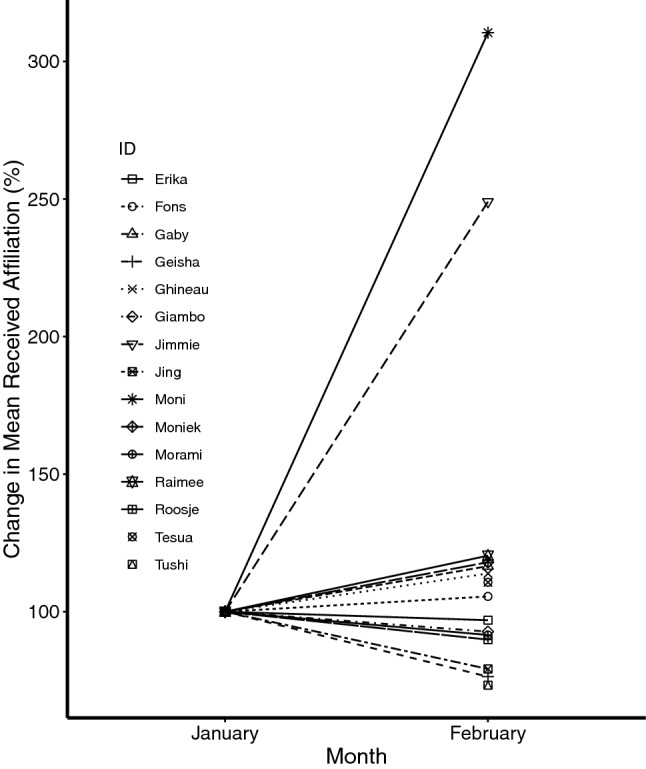
Percentage change in mean hourly rates of affiliation received by each individual in the month before (January) and after (February) the death of the infant as predicted by the model, using January as 100%

A closer examination of the change in predicted received affiliation in the month directly post-event compared to the month directly pre-event shows that the magnitude of difference was much stronger for Moni than for any other individual in the group (Fig. 3). Only one other individual, Jimmie, showed a similar notable increase.

**Fig. 4:**
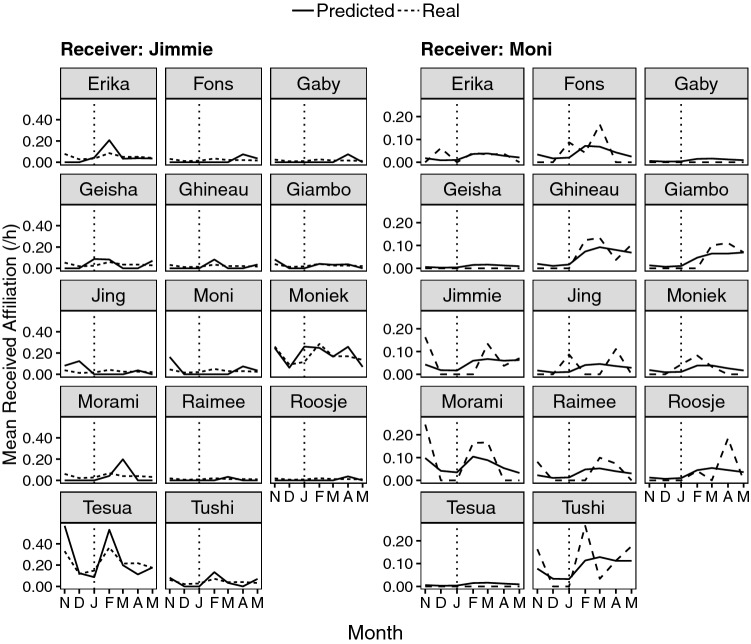
Actual and model estimate of mean hourly rate of affiliation directed towards Moni and Jimmie by each individual in the group per month. The vertical line indicates the stillbirth of the infant

As Fig. 4 illustrates, various partners drove the reported patterns for Moni, with individual differences. For example, five individuals (Erika, Ghineau, Morami, Roosje, and Tushi) who showed no affiliation with Moni in the month pre-event started affiliating with her in the month post-event. The number of individuals changing from no affiliation to affiliation was unparalleled among the group members (modus = 1 individual; median = 2 individuals; mean = 2.14 individuals, see Online Resource 2, Table 5). The steepest increases in Moni’s received affiliation rate were attributable to Tushi (0.27/h in February), then Morami (0.16/h), and Ghineau (0.12/h), all at least 1.5 times higher than the group mean of received affiliation rates in this month (0.08/h). Changes of such high magnitude (i.e., higher than the group mean) rarely occurred for other group members (modus = 0 individuals, median = 0 individuals, mean = 0.54 individuals). Jimmie’s rise in received affiliation was driven predominantly by Moniek and Tesua (Fig. 4).

### Qualitative data

Descriptive analyses of reassurance behavior revealed that Moni never engaged in a mouth–mouth kiss across the entire observation period, except for the day following the event, when she was the recipient and actor of several instances with two males (Online Resource 1). Furthermore, on the day of the event itself, Moni received six body kisses, a rarely occurring behavior during the observation period (13 instances over ± 130 observation hours). Another behavior that differed notably before, during, and after the event was finger/hand in mouth. Whereas Moni never received this behavior in the pre-period (± 43 observation hours), she received it four times on the day of the event, and three times in the post-period (± 87 observation hours).

## Discussion

In this case report, we show that chimpanzees temporarily increased the quantity and quality of their affiliative behaviors towards a (recently) bereaved mother. In particular, we identified a selective and marked peak in affiliation towards the bereaved mother immediately following her infant’s death, while simultaneously accounting for alternative factors (discussed below) that might explain such an increase. Furthermore, the highest rates of affiliation came from individuals who had not been observed to affiliate with the bereaved mother at all in the month prior to the event. These data strongly suggest that many of the chimpanzees in the study group engaged in a form of consolatory behavior toward the bereaved mother.

It is noteworthy that behaviors with putative reassurance functions were directed to the newly bereaved mother, though these behaviors were rarely if ever observed outside of this context. Such behaviors (e.g., body kisses, mouth–mouth contacts, and finger/hand in mouth) are characteristic of reconciliation and consolation contexts, the latter often considered a key marker of empathy (Preston and de Waal 2002) given its putative role in stress alleviation (Fraser et al. 2008; Romero and de Waal 2010; cf. Koski and Sterck 2007).

In human societies, tending of the dying and the treatment of the bereaved are typically governed by empathetic behavior, including increased targeted affiliation during and after such distressing events (Sinclair et al. 2016; Aoun et al. 2018; Bonanno 2009). In chimpanzees, however, these contexts are still understudied—partly due to the impossibility of applying an experimental approach, but also from an underappreciation of what we can learn from death-related behaviors in other species (Cronin et al. 2011; Stewart et al. 2012). Moreover, chimpanzees’ tendency to console others in distress has been overwhelmingly studied in the context of social aggression (i.e., post-conflict contexts), wherein bystanders offer affiliation towards recent conflict victims in the minutes following these distressing events (reviewed in de Waal and Preston 2017). Responses to death-related events therefore represent an untapped source of potential knowledge regarding consolation and (other forms of) empathetic concern. Overall, in combination with our more systematic quantitative data, qualitative observations around the event suggest that chimpanzees increase affiliation towards a bereaved mother by means of empathetic responses. The present results suggest that subsequent to other distressing events, behavioral expressions of empathy may be evident over extended periods of time, rather than occurring only in the minutes or hours following an event.

Though not necessarily incompatible with an empathy hypothesis, it is also possible that the rise in reassurance behaviors surrounding the event was partially attributable to increased tensions (e.g., due to Tushi holding the infant, or the more confined space in the backstage enclosures). Further, because conciliatory behaviors may also function to relieve distress in bystanders of aggression (Fraser et al. 2009), it is possible that increases in affiliation could be due to overall increased stress levels in the group, though we did not observe similar affiliative changes for all group members. In the present study, it remains ambiguous to what extent the bereaved mother solicited the increased affiliation she received from other group members post-event. Future research could examine whether behavioral or physiological indicators of stress prompt individuals to affiliate with the bereaved. Regardless of the precise mechanism, the increase in received affiliation could have buffered the bereaved mother against the stressful consequences of her loss (for an elaborate example in baboons, see Engh et al. 2006), which corroborates the suggested function of consolation in chimpanzees (de Waal and Preston 2017).

We explored several alternative hypotheses for the augmented expressions of affiliation towards the bereaved mother. First, we found no significant difference in received affiliation when the bereaved mother was swollen compared to not swollen. Furthermore, the bereaved mother only showed her first swelling post-event 2 months later. However, as expected on the basis of prior studies (Nishida 1979; Wallis and Lemmon 1986), the bereaved mother received comparatively little affiliation when she was pregnant. Although parturition coincided with the event, making it possible that changes in her received affiliation were attributable to corresponding changes in reproductive state, the aforementioned studies reveal that diminished affiliation towards pregnant females was specific to interactions with male partners (Nishida 1979; Wallis and Lemmon 1986). In the present study, females were largely responsible for the increased affiliation towards the bereaved mother. Together with the magnitude of the observed increase (210%)—much larger than the ± 100% increase in received affiliation described in previous research on pregnant females (Wallis and Lemmon 1986, Table 1)—this leads us to tentatively conclude that pregnancy effects were not solely responsible for the changes in affiliation following the event.

Second, we explored whether a natural integration process could account for the observed changes in affiliation over time. There are several lines of counter-evidence worth noting. By including the other group members in the analysis, we determined that the curve of the bereaved mother’s received affiliation was different from that of another newly introduced female (Erika). Indeed, received affiliation trajectories showed no clear evidence of a group stabilization process finalizing in the month(s) following the event (i.e., there was no uniform rise in affiliation followed by a plateau). Further, aggression rates towards Moni and Erika remained relatively stable across the study period, as did the amount of support they received in conflicts.

Third, progressively during the study period, subjects were observed more in the outdoor than indoor enclosure. However, observation location was not a significant predictor of the affiliation received by Moni, nor by the rest of the group. Lastly, there was an additional possibility that affiliative responses towards Moni were indirectly driven by curiosity towards the dead infant, rather than by a direct motivation to affiliate with the bereaved mother. However, given that the corpse was not present in any of the observations used in the model, we can rule out this possibility.

The marked increase in received affiliation by the bereaved mother was only paralleled, albeit to a lesser extent, by one other group member, Jimmie, an effect attributable to two females, Tesua and Moniek. As these two females are kin (sister and daughter, respectively) of the two females responsible for the strongest increases in the bereaved mother’s received affiliation (i.e., Tushi and Morami), it is conceivable that Tesua and Moniek redirected their affiliation towards Jimmie because their usual affiliation partners were unusually invested in the bereaved mother. In any case, this provides a further illustration of how changed behaviors (at the individual level) towards the bereaved may cascade through the group.

While many group members increased affiliation towards Moni following the loss of her infant, some patterns were more notable than others, possibly reflecting various relational (e.g., the quality of the social bond), individual (e.g., personal history and experience), and circumstantial factors. Tushi’s pronounced increase in affiliation towards Moni is interesting given Tushi’s personal history, which includes having a stillborn infant in 2004; conceivably, this might have boosted compassionate tendencies towards Moni, or at least motivated heightened interest in the corpse. Another possibility is that this shared experience served to augment Tushi’s own stress response to the event. In humans, previous experiences with grief may boost empathy for the suffering of others, particularly those in similar situations (Lichtenthal et al. 2010). While we acknowledge the general limitations of case reports (Sarringhaus et al. 2005), we suggest that the possible link between shared experiences and consolatory responses is worthy of future study in the general disciplines of thanatology and comparative psychology.

## Conclusions

This exploration of a group response to the death of an infant and the bereaved mother provides insights into how chimpanzees deal with intense, socially disruptive events such as death, and how they respond to the individuals most closely affected it. Overall, our observations provide initial indications that chimpanzees may extend their attention from the deceased to the bereaved in the form of temporarily increased affiliation. These findings broaden the scope for chimpanzees’ consolatory capacities, call for further study of death-related responses in non-human animals to include larger-group dynamics rather than individual responses to the deceased, and further elucidate the possible breadth of the evolutionary roots of human behavior.

## Electronic supplementary material

Below is the link to the electronic supplementary material.

Supplementary material 1 (PDF 67 kb)

Supplementary material 2 (PDF 181 kb)

## Data Availability

The data sets collected and/or analyzed during the current study are available from the corresponding author on reasonable request.

## References

[CR1] Anderson JR, Gillies A, Lock LC (2010). *Pan* thanatology. Curr Biol.

[CR2] Anderson JR, Biro D, Pettitt P (2018). Evolutionary thanatology. Philos Trans R Soc B Biol Sci.

[CR3] Aoun SM, Breen LJ, White I, Rumbold B (2018). What sources of bereavement support are perceived helpful by bereaved people and why? Empirical evidence for the compassionate communities approach. Palliat Med.

[CR4] Baayen RH (2008). Analyzing linguistic data.

[CR5] Barr DJ, Levy R, Scheepers C, Tily HJ (2013). Random effects structure for confirmatory hypothesis testing: keep it maximal. J Mem Lang.

[CR6] Biro D, Humle T, Koops K, Sousa C, Hayashi M, Matsuzawa T (2010). Chimpanzee mothers at Bossou, Guinea carry the mummified remains of their dead infants. Curr Biol.

[CR7] Boesch C, Boesch-Achermann H (2000). The chimpanzees of the Taï forest: behavioural ecology and evolution.

[CR8] Bonanno GA (2009). The other side of sadness: what the new science of bereavement tells us about life after loss.

[CR9] Cronin KA, van Leeuwen EJC, Mulenga IC, Bodamer MD (2011). Behavioral response of a chimpanzee mother toward her dead infant. Am J Primatol.

[CR10] de Waal FBM (2014). The bonobo and the atheist: In search of humanism among primates.

[CR11] de Waal FBM, Preston SD (2017). Mammalian empathy: behavioural manifestations and neural basis. Nat Rev Neurosci.

[CR12] de Waal FBM, van Roosmalen A (1979). Reconciliation and consolation among chimpanzees. Behav Ecol Sociobiol.

[CR13] Dobson AJ (2002). An introduction to generalized linear models.

[CR14] Engh AL, Beehner JC, Bergman TJ (2006). Female hierarchy instability, male immigration and infanticide increase glucocorticoid levels in female chacma baboons. Anim Behav.

[CR15] Forstmeier W, Schielzeth H (2011). Cryptic multiple hypotheses testing in linear models: overestimated effect sizes and the winner’s curse. Behav Ecol Sociobiol.

[CR16] Fraser ON, Aureli F (2008). Reconciliation, consolation and postconflict behavioral specificity in chimpanzees. Am J Primatol.

[CR17] Fraser ON, Stahl D, Aureli F (2008). Stress reduction through consolation in chimpanzees. Proc Natl Acad Sci USA.

[CR18] Fraser ON, Koski SE, Wittig RM, Aureli F (2009). Why are bystanders friendly to recipients of aggression?. Commun Integr Biol.

[CR19] Goodall J (1989). Glossary of chimpanzee behaviours.

[CR20] Kooriyama T (2009). <Note > The death of a newborn chimpanzee at Mahale: reactions of its mother and other individuals to the body. Pan Afr News.

[CR21] Koski SE, Sterck EHM (2007). Triadic postconflict affiliation in captive chimpanzees: does consolation console?. Anim Behav.

[CR22] Lichtenthal WG, Currier JM, Neimeyer RA, Keesee NJ (2010). Sense and significance: a mixed methods examination of meaning making after the loss of one’s child. J Clin Psychol.

[CR23] Martin P, Bateson P (2007). Measuring behaviour.

[CR24] Nishida T, Hamburg DA, McCown ER (1979). The social structure of chimpanzees of the Mahale Mountains. The great apes.

[CR25] Payne EH, Gebregziabher M, Hardin JW, Ramakrishnan V, Egede LE (2018). An empirical approach to determine a threshold for assessing overdispersion in Poisson and negative binomial models for count data. Commun Stat Simul Comput.

[CR26] Preston SD, de Waal FBM (2002). Empathy: its ultimate and proximate bases. Behav Brain Sci.

[CR32] R Core Team (2018) R: a language and environment for statistical computing. R Foundation for Statistical Computing, Vienna, Austria. https://www.R-project.org/. Accessed 7 May 2019

[CR27] Romero T, de Waal FBM (2010). Chimpanzee (*Pan troglodytes*) consolation: third-party identity as a window on possible function. J Comp Psychol.

[CR28] Sarringhaus LA, McGrew WC, Marchant LF (2005). Misuse of anecdotes in primatology: lessons from citation analysis. Am J Primatol.

[CR29] Schel AM, Rawlings B, Claidière N (2013). Network analysis of social changes in a captive chimpanzee community following the successful integration of two adult groups. Am J Primatol.

[CR30] Sinclair S, Norris JM, McConnell SJ (2016). Compassion: a scoping review of the healthcare literature. BMC Palliat Care.

[CR31] Stewart FA, Piel AK, O’Malley RC (2012). Responses of chimpanzees to a recently dead community member at Gombe National Park, Tanzania. Am J Primatol.

[CR33] Teleki G (1973). Group response to the accidental death of a chimpanzee in Gombe National Park, Tanzania. Folia Primatol.

[CR34] van Leeuwen EJC, Mulenga IC, Bodamer MD, Cronin KA (2016). Chimpanzees’ responses to the dead body of a 9-year-old group member. Am J Primatol.

[CR35] Wallis J, Lemmon WB (1986). Social behavior and genital swelling in pregnant chimpanzees (*Pan troglodytes*). Am J Primatol.

