# Supplementary material for: Do chimpanzees (Pan troglodytes) console a bereaved mother?
Source: Primates. 2019 Sep 4;61(1):93–102. doi: 10.1007/s10329-019-00752-x (PMC6971188; doi:10.1007/s10329-019-00752-x)
Supplement: Supplementary file 1 — Supplementary material 1 (PDF 67 kb) [file 10329_2019_752_MOESM1_ESM.pdf]

Do chimpanzees (*Pan troglodytes*) console a bereaved mother?, Primates, Zoë Goldsborough[1]*, Edwin J. C. van Leeuwen, Kayla W. T. Kolff, Frans B. M. de Waal, Christine E. Webb

[1] Department of Biology, Utrecht University, Utrecht, the Netherlands

[*] Corresponding author: zgoldsborough@outlook.com

Online Resource 1 – Description of events surrounding stillbirth of infant

On the day of the event (30-01-2018), feeding took place at the same time as Moni and the infant's corpse rejoined the group. Afterwards, Moni sat in a nest on her own with the infant's corpse and was approached by several individuals, who initiated affiliative behaviors such as hand-in-mouth, body-kiss and grooming. The two individuals who were most often near Moni and the infant's corpse were Fons and Tushi, followed by the alpha male Giambo. Moni interacted with the corpse in various ways, including inspecting, grooming, and manipulating it (i.e., lightly slapping its chest, biting the umbilical cord, and lifting the corpse upside down and sweeping it across the floor). Initially, when other individuals would try to touch the infant, Moni would pull it away or move to another compartment. Later in the day, she did allow other individuals to inspect and groom the infant, but she generally remained within 1-2 meters of the infant at all times. No agonistic interactions were observed during this observation period, aside from some submissive greetings by Moni and other group members, mostly towards the alpha male (which is

noteworthy given the tendency for conflict in the backstage enclosures, which are considerably smaller than the other holding areas).

On 31-1-2018, no observations were conducted, but the chimpanzees remained in the backstage enclosures. On the following day (01-02-2018), the infant's corpse was picked up by Tushi in the morning before the observers arrived. Later that morning, when Tushi and the infant's corpse were separated from the rest of the group, Tushi was observed engaging closely with the infant's corpse. For several hours, she ignored food. She groomed and inspected the corpse (seemingly more than Moni had done) and kept it against her body for most of the time. While Tushi was separated from the group, we observed Moni and Fons displaying and banging on the hatch leading to the backstage enclosures on multiple occasions. Later that day, Moni, Fons and the beta male, Jing, entered Tushi's enclosure. Moni appeared anxious and behaved submissively towards Tushi, baring teeth and fear-screaming, while extending her hand toward the males. She received several affiliative gestures such as mouth-mouth kisses, body-kisses and finger/hand-in-mouth from Fons and Jing. Tushi displayed at Moni, while holding and swinging the corpse. At one point, Tushi sat down on a tire above Moni's head, and dangled the infant's corpse above Moni, pulling it out of reach when she tried to grab it. This interaction was repeated several times until the rest of the group was let into the enclosures. One female, Gaby, approached Tushi to inspect the infant, whereupon Tushi repeatedly pushed the infant's corpse against Gaby's face in a forceful manner.

Three days after the event (02-02-2018), when Tushi returned to the group without the corpse, several individuals gathered around her to smell and inspect her. Tushi was

observed on the periphery of the group for much of the day, despite being among the most socially integrated members. On one occasion, Tushi approached the rest of the group, who were all sitting on one platform. When she neared the others, Moni displayed at her and slapped her, Tushi went to the alpha male and touched him, while the beta male abandoned his grooming partner to groom Moni. Several days later, Tushi and Moni were seen to spend a lot of time mutual-grooming, and showed no further signs of agonism.
