# Supplementary material for: Do chimpanzees (Pan troglodytes) console a bereaved mother?
Source: Primates. 2019 Sep 4;61(1):93–102. doi: 10.1007/s10329-019-00752-x (PMC6971188; doi:10.1007/s10329-019-00752-x)
Supplement: Supplementary file 2 — Supplementary material 2 (PDF 181 kb) [file 10329_2019_752_MOESM2_ESM.pdf]

Do chimpanzees (*Pan troglodytes*) console a bereaved mother?, Primates, Zoë Goldsborough[1]*, Edwin J. C. van Leeuwen, Kayla W. T. Kolff, Frans B. M. de Waal, Christine E. Webb

[1] Department of Biology, Utrecht University, Utrecht, the Netherlands

[*] Corresponding author: zgoldsborough@outlook.com

## Online Resource 2 – Supplemental results

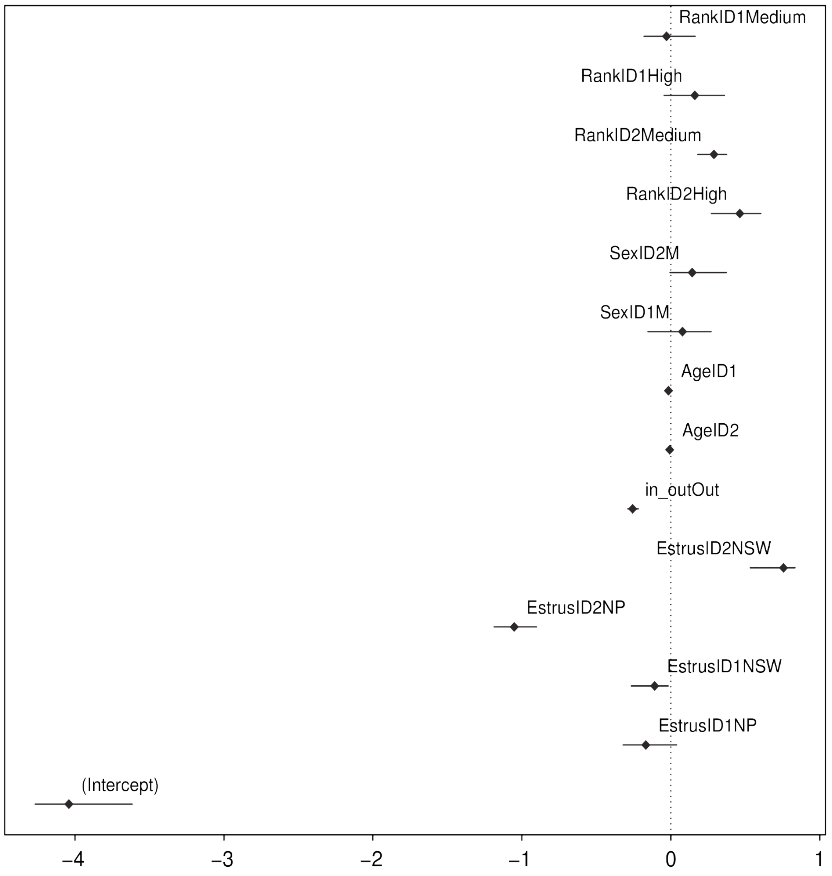


**Figure 5 – Model stability of the group-level analysis.** Lines indicate the range of parameter estimates based on sequential removal (with replacing) of subjects and re-running the model. The obtained estimate ranges are judged to be reasonable and expected given the relatively small sample size (n=15).

**Table 3 – Generalized Linear Mixed Model output of the group-level analysis**

| Effect | $\chi^2$ | df | P | Estimate ±SD (P) |
|---|---|---|---|---|
| Intercept | [a] | [a] | [a] | -4.378 ± 0.575 |
| Age Actor | 6.642 | 1 | 0.010** | -0.026 ± 0.009 |
| Age Recipient | 0.274 | 1 | 0.610 | -0.004 ± 0.008 |
| Estrus Actor (ref: not swollen) | 1.514 | 2 | 0.469 | Pregnant: -0.194 ± 0.168 (0.248) Swollen: -0.050 ± 0.082 (0.542) |
| Estrus Recipient (ref: not swollen) | 127.770 | 2 | <0.001*** | Pregnant: -0.812 ± 0.321 (0.011) Swollen: 0.757 ± 0.068 (<0.001) |
| In/out (ref: In) | 2.136 | 1 | 0.144 | -0.264 ± 0.179 |
| Rank Actor (ref: Low) | 1.097 | 2 | 0.578 | Medium: -0.170 ± 0.250 (0.497) High: -0.344 ± 0.323 (0.287) |
| Rank Recipient (ref: Low) | 0.727 | 2 | 0.695 | Medium: 0.192 ± 0.235 (0.413) High: 0.205 ± 0.297 (0.491) |
| Sex Actor (ref: Female) | 2.375 | 1 | 0.123 | 0.471 ± 0.293 |
| Sex Recipient (ref: Female) | 2.588 | 1 | 0.108 | 0.432 ± 0.266 |

[a] Not indicated because of having a limited interpretation

**Table 4 – Overview of incidences of contact aggression.** Both initiated by and directed at Moni and Erika per month of the study period, including when the amount of instances that they received support (i.e. another individual joins the conflict on their behalf)

| | **Moni** | | | **Erika** | | |
|---|---|---|---|---|---|---|
| **Month** | #Victim | #Aggressor | #Support | #Victim | #Aggressor | #Support |
| **N**ov | 3 | 0 | 1 | 1 | 0 | 0 |
| **D**ec | 1 | 0 | 0 | 3 | 2 | 1 |
| **J**an | 2 | 1 | 0 | 3 | 1 | 0 |
| **F**eb | 9 | 2 | 2 | 0 | 0 | 0 |
| **M**ar | 3 | 0 | 1 | 2 | 2 | 0 |
| **A**pr | 1 | 0 | 0 | 0 | 1 | 0 |
| **M**ay-Jun* | 2 | 0 | 2 | 1 | 2 | 0 |

* Only one day was observed in June

**Table 3. Selective overview of affiliation patterns during the immediate months pre- and post-event.** For each receiver of affiliation, we counted how many previously non-affiliating group members started affiliating in the post-event month, and how many of them assumed an affiliation rate higher than the mean affiliation rate in the post-event month (0.08/h).

| **Receiver** | **Non-affiliating to affiliating (in individuals)** | **New affiliation rate > mean (0.08/h)** | **Names of the actors of > mean increase** |
|---|---|---|---|
| Erika | 4 | 0 | |
| Fons | 3 | 1 | Erika |
| Gaby | 4 | 1 | Moni |
| Geisha | 1 | 0 | |
| Ghineau | 2 | 1 | Tesua |
| Giambo | 0 | 0 | |
| Jimmie | 4 | 1 | Tushi |
| Jing | 2 | 0 | |
| Moni | 5 | 3 | Ghineau, Morami, Tushi |
| Moniek | 2 | 0 | |
| Morami | 3 | 2 | Erika, Moni |
| Raimee | 1 | 0 | |
| Roosje | 2 | 1 | Jing |
| Tesua | 1 | 0 | |
| Tushi | 1 | 0 | |
